# Marriage stability in a pastoralist society

**DOI:** 10.1093/beheco/arz115

**Published:** 2019-07-08

**Authors:** Juan Du, Ruth Mace

**Affiliations:** 1 State Key Laboratory of Grassland and Agro-ecosystems, School of Life Sciences, Lanzhou University, Tianshui Nan Lu, Lanzhou, Gansu Province, PRC; 2 Department of Anthropology, UCL, London, UK

**Keywords:** Amdo, brideprice, divorce, dowry, labor, marriage, trial marriage, wealth

## Abstract

We examined how individual investment was associated with the duration of marriage partnerships in a pastoralist society of Amdo Tibetans in China. We collected demographic and socioeconomic data from 420 women and 369 men over five villages to assess which factors predicted partnership length. We found that the payment of dowry and bridewealth from both sides of the family predicted marriage stability. The production of offspring, regardless of their survivorship, also had a positive effect on marriage duration, as did trial marriage, a time period before formal marriage. Finally, we found that if both bride and groom invest resources initially into a partnership—whether wealth or labor—their subsequent partnership is stronger than couples who do not make such investments. This paper adds to our understanding of complex social institutions like marriage from a behavioral ecological perspective.

## Teaser

Choosing a long-term partner is literally a life-changing decision. Here, using data from Tibetan pastoralists, we found that a trial marriage, which gives both parties the opportunity to assess the other’s quality, resulted in more stable marriages. The qualities assessed include female labor ability before marriage, brideprice payment at marriage, and fertility after the marriage. We also found that if either or both sides of the partnership invested wealth into the marriage, the duration of the marriage was longer than when partners did not make such contributions.

## INTRODUCTION

The institution of marriage is almost universal across human societies, and marriage norms generally proscribe the responsibilities of caring for children. Nevertheless, the stability of marriages across cultures is highly variable, with reasons for divorce varying across different ecologies (South et al. 2001; Sear et al. 2003; Anderson et al. 2007; Jalovaara 2013). Individuals are more likely to leave a marriage when the benefits are reduced and the cost of leaving is low. In evolutionary terms, divorce can be seen as a trade-off between the welfare of children produced within the existing marriage and the future mating opportunities that might lead to further reproductive success with a new partner. In rural Gambia, for example, divorce is common when there are no offspring, and women formally marry only after they have produced one or two children in their natal home (i.e., after providing evidence of their fertility) (Sear et al. 2003). Infant mortality can also result in women seeking a divorce: in Bangladesh, for example, divorce is associated both with child mortality and family abuse (Nag 1972; Alam et al. 2016). In some societies, child mortality risk is increased among divorced families because resource provisioning from the father is important for child survival (Marlowe 2000), suggesting that variation in parental investment may be a key factor. A clear constraint on the divorce process is concerns over which party will take care of the children following marital dissolution (Weisner and Gallimore 1977). Disinvestment can also be triggered by a tense marriage relationship if a man pursues self-serving activities at the cost of his partner’s or family’s resources (Stieglitz et al. 2011, 2012).

Wealth and education are also important contributory factors to marital stability (Amato 2010). Wealth transfers at marriage are common in many societies, especially traditional farming and herding societies. Marriage payments can be divided into a number of different forms: bridewealth, dowry, bride-service, gift-exchange, and token (Goody and Tambiah 1973; Bossen 1988). Polygyny and pastoralism often coevolve with bridewealth (transfers from the groom or his family to the bride’s family) as a form of male-biased parental investment aimed at enhancing a son’s chances of becoming polygynous (Hartung et al. 1982). In contrast, dowry refers to a payment that the bride’s family makes to the groom’s family or to the marrying couple at the time of marriage: a form of female-biased property inheritance that tends to be practiced in societies with land property and mainly with monogamy. Gaulin and Boster (1990) proposed a “female-competition model” arguing that, in stratified and monogamous societies, dowry is used as a means of competition among women for desirable husbands. In ancient China, dowry served to distinguish the higher status wife from the concubine (Ebrey 1986; Fortunato et al. 2006). Large dowries are also common in India and China under conditions where hypergyny is almost impossible.

Siblings can also play a role in the provision of bride wealth and dowry, because they compete for the family property, and this can have downstream consequences for marital stability. In pastoralist societies like the Gabbra, for example, number of brothers is positively associated with the age of male marriage, the number of livestock given to start the new household, and the size of dowry provided to daughters (Mace 1996). Siblings not only compete for the family resources, however, but may also collaborate to protect the family property and maximize reproductive interests (Ji et al. 2014, 2013). Sororal polygyny and fraternal polyandry are examples of siblings cooperating by staying in the same marriage. Kin selection theory predicts that, in polygynous marriages, genetically related sisters should show less conflict than cowives that lack genetic relatedness (Chisholm and Burbank 1991; Winking et al. 2007; Winking et al. 2013). The same applies to brothers in polyandrous marriages. Fraternal polyandry is also practiced as a functional response to the dynamics of wealth transmission over time, as seen in the Himalayas (Childs 2003). It is often the case that younger siblings will leave polyandrous marriages and find other partners if and when additional economic opportunities arise (Haddix and Gurung 1999; Haddix 2001; Levine and Silk 2008).

Personal characteristics are also crucial to marriage stability. In one African matrilineal society, for example, lazy men are often required to desert the marriage under the maternal uncle’s orders (Kishindo 2011). In contrast, indications of good character can increase marriage ability and the stability of unions, for example, being good at housework, higher levels of education (Gibson and Lawson 2014), or knowledge (Tzeng 1992), wealth, skilled story-telling ability (Smith et al. 2017), and physical attractiveness (Pedersen 1991). Age of marriage (Bumpass and Sweet 1972), provision of child support (Cherlin 1977), religious divergence (Bumpass and Sweet 1972), marginal benefits of staying (Amato 2000; Blurton et al. 2000; Amato 2010), and sex ratio (Uggla and Andersson 2018) also affect marital stability.

Theoretically, it has also been shown that the level of investment placed into a given relationship can affect marital commitment (Rusbult 1980), but this has not been tested in a real-world context. Similarly, Gurven and Hill (2009) argue that there should be a dynamic equilibrium in terms of family investment: if one party invests less, the other party has to invest more to strike an acceptable balance, and divorce will occur if such “bargaining” fails. While other family members can help with childcare and other aspects of family life (Sear and Mace 2008), marriage partners play the most crucial role in stabilizing a relationship via investment into the family unit, whether this is through labor and/or wealth. Wealth investment is mostly exhibited in the inheritance and marital payment/bridewealth system within a family, especially in the farming and pastoralist societies, where wealth is in the form of land or livestock.

Although several demographic studies have investigated the reasons underlying marital stability and dissolution, there have been very few attempts to explain it from a behavioral ecological perspective. Here, we investigate the behavioral ecology of divorce within the ethnographic context of a pastoralist society in the Maqu region of Tibet. The marital system in our study population includes polygamy (both polygyny and polyandry), but is predominately monogamous. This is partly because wealth disparities and social hierarchies are rather weak, and also because the presence of multiple wives or husbands is now treated as representing a “backward lifestyle” in the national administration’s point of view. The pastoralist lifestyle in the region remains mobile. In general, the herders occupy two sites over the course of a year: one summer site, in more remote high-altitude areas where families live in yurts (yak hair tents), and a winter site, which is more settled, more accessible to local towns and comprises of houses built of mud or bricks. Traditionally, herders moved between many more sites each year contingent on the condition of the grassland and water supply.

Another feature of our study population is trial marriage, where men and women cohabit before formal marriage. In Tibetan society, the mating system is quite relaxed before cohabitation, in that men and women may have multiple sexual partners. Once the decision is made to live together, however, mate switching is much less common, and mate guarding by men appears to be stronger. In the past, partner selection was more likely to be arranged by parents or relatives, but with the development of modern communications, such as mobile phones, young people have more freedom to contact each other directly; the parents’ opinions remain crucial, however. A trial marriage is usually entered into after a couple have seen each other on several occasions. Parents can also arrange a marriage for their children, and in this case, the entire process becomes simpler as the couple often skips the trial period.

**Figure 1:**
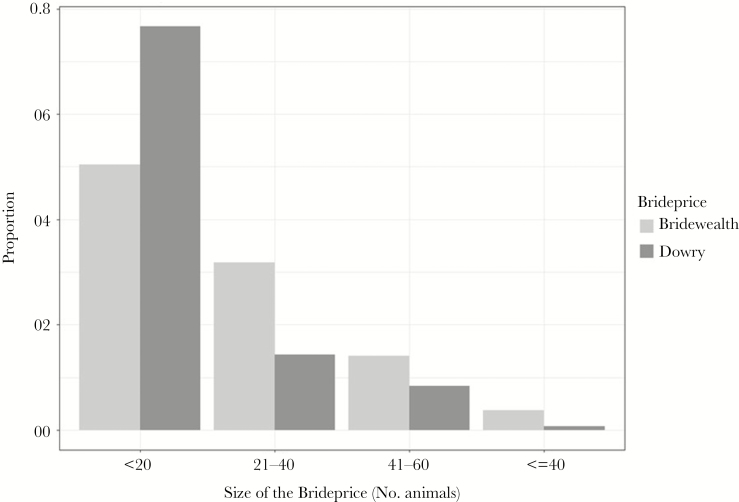
Distribution of bridewealth in number of yaks. The x-axis is the size of the bridewealth, the y-axis is proportion of marriages where bridewealth or dowry is given. Light gray indicates bridewealth; dark gray is dowry.

In Maqu, both bridewealth and dowry are practiced regardless of the postmarital residence pattern (Figure 1); bridewealth is called *jerrah* (རྒྱུ་རིན) and dowry is called *Wahe jiong* (བག་རྫོངས). From the 1950s to 1980s, both bridewealth and dowry were rare, because no private property was allowed by the Communist administration (the commune system) (Du and Mace 2018). Instead, everyone in a household was allocated very few yaks. Upon marriage, each person would bring his/her own property from his/her natal home to form the new household (Gelek and Miao 2002). In the 1980s, marriage payments resumed, and are paid before or on the day of marriage from the man’s family to the woman’s family or to the new couple. The nature of bridewealth varies but, in general, it mainly consists of yaks, alongside cash, tea, grain, and butter. In the case of dowry, it mainly consists of yaks, expensive jewellery (coral necklaces, silver belts, gold earrings, etc.), and sheepskin garments. Both sets of parents traditionally provide half of the yak hair tent for the new couple. All the yaks that make up the bridewealth and dowry remain with the couple and, in the event of divorce, whomever leaves the house is entitled to take the full bridewealth or dowry back with them. Both women and men have the right to end a marriage (see more details of the ethnographic information in Supplementary Information).

Temporal and geographical differences in operational sex ratio can be an important predictor of pair-bond stability (Blurton et al. 2000; Uggla and Andersson 2018). A female-biased sex ratio should make remarriage after divorce easier for men. As we are only studying one population, we cannot examine the effects of sex ratio on divorce, but we present data on the sex ratio to illustrate the context of divorce decisions in this population. Considerations of sex ratio are relevant because sending at least one son from each extended family to the monastery is a tradition in Tibetan culture. At some points in Tibetan history, up to a third of males were in the monastery for most of their life. With the implementation of the child policy in the late 1980s and compulsory education, however, the size of the monasteries was reduced and the number of monks and nuns declined accordingly (Hao 2000). Abortion or differential investment in the less preferred sex in some parts of China can also lead to a biased population sex ratio (Hesketh and Xing 2006).

Based on the above theoretical considerations and the particular circumstances of our study population, we predicted: 1) the presence of children will have a positive impact on marriage stability; 2) that payment of either bridewealth or dowry will increase marital stability; and 3) that the presence of a “trial marriage” will reduce the chances of subsequent divorce, as this offers potential marital partners a period of assessment prior to a binding commitment with respect to the sharing of labor and other responsibilities within a partnership and 4) that a high reputational capacity for labor (as a potential measure of investment in the household) will be associated with a reduced likelihood of divorce. Thus, female fertility, wealth investment and labor ability are all predicted to increase marriage security, providing a pathway to understanding partner choice decisions in a real-world setting.

## METHODS

### Study site

Field work was conducted in Maqu, an administrative district in the southwest region of Gannan Tibetan autonomous prefecture in Gansu province, China. Our research was approved by the UCL Research Ethics committee and Lanzhou University. Maqu is a part of Amdo Tibet and lies at the intersection of Qinghai, Gansu, and Sichuan provinces. Maqu spans an area of about 10,190 km^2^ and is home to 54,900 people according to a census in 2011. Average altitude of Maqu reaches 3500~3800 m above sea level with an average temperature of 1.2 ℃ across the year and annual average rainfall of 611.9 mm (information from Maqu records). The largest indigenous population is “Brog pa,” herders making their living from selling yaks and sheep; some of them also receive government benefits as a supplementary income. Maqu, named after the Yellow River that runs through the County, is made up of one town and seven “Xiang” (or rural townships. Nima town (meaning sun in Tibetan), is the political, cultural, and economic center of Maqu with several surrounding villages. People in Maqu share a common culture and speak a distinctive dialect of Tibetan (Levine 2014).

**Figure 2:**
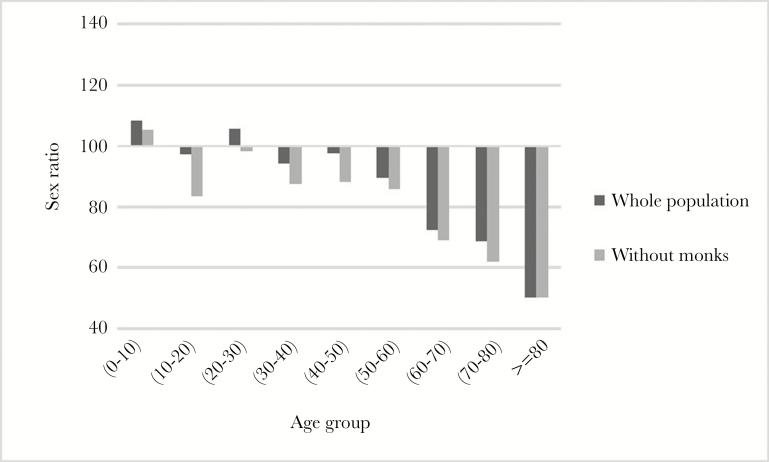
Sex ratio in the population by 10-year age groups. Black bar indicates the sex ratio of the whole population, and gray bar indicates operational sex ratio excluding monks. Numbers over 100 indicate more males than females in the population.

There are 696 households in five villages in our study site. In 2015, there were 182 living monks and 15 nuns in our study villages. The operational sex ratio excluding monks for the population is 89.83 (Supplementary Table 1), that is, it is significantly skewed towards females (chi square = 12.54, *P* = 0.001, 95% confidence interval [CI] = 0.46, 0.49). Figure 2 shows the distribution of operational sex ratio in different age groups. After 35 years of age, the sex ratio is female-biased. The skewed sex ratio is especially pronounced in older age groups (Supplementary Table 1 shows the sex ratio with and without monks in this society). The mean age of monks in the population is 29.14 (min age = 7.00, max age = 80.00, SD = 15.89). We used the age range between 15 and 50 to calculate the operational sex ratio (Coxworth et al. 2015; Uggla and Mace 2016). The operational sex ratio is 98.82 in the whole sample and 90.48 excluding monks. The latter more closely reflects the level of intrasexual competition because monks are not in the mating market. As the size of monasteries has been declining in the last few decades, it is possible that the operational sex ratio may have been even more female-biased in the past.

### Data collection

Demographic data on births, deaths, family composition, and marriages of all household members were collected by J.D., with the help of local research assistants, via in-depth interviews between June–October 2014 and March–December 2015. In each interview, we collected information through a household census, which included demographic data on all household members and the household economy taken from the head of the household; we also interviewed both men and women separately in private spaces about their marriage history. This included information on any previous marriage partners, the length of any trial marriage, the amount of bridewealth and dowry, start and end year of each relationship, what happened after divorce (e.g., how was the bridewealth payment dealt with), whether any children has been produced during the marriage, and the year of birth and current survival status of the offspring. Seven hundred and eighty-nine individuals’ marriage history data were included in the analysis (male = 369, female = 420). Some people divorced more than once (Supplementary Figure 1 for marital status), but we look only at first marriage history for both men and women. There was no difference between males and females regarding most of the predictors of divorce (Supplementary Table 4). In order to avoid any possible double counting, we focus only on women in our analyses.

We chose three senior female villagers (mean age = 47.33 ± 3.06 SD), with knowledge of everyone in the village, to rate the working ability of women. They rated the women in their village (*n* = 186) on working ability on a scale of 0 to 3 with 0 being “not hard working” and 3 being “good at working” (see Figure 4 for rating result). The women being rated were individuals from whom we had collected detailed demography data, and we made a name card with their picture so that it was easy for the rater to recognize them. To reduce the risk of biased opinions, raters could communicate among themselves while rating, in order to reach a jointed agreement on the score. One hundred and eighty-six female subjects’ rating data were used in this analysis (min age = 19, max age = 76, mean age = 38.62 ± 12.38 SD; see Supplementary Figure 2 for the age distribution of the sample).

### Statistical analyses

Event history analysis was used to examine the determinants of time to divorce in women. Event history analysis models the duration of time until an event of interest occurs (Allison 1982). This method enables us to include both time-varying and time-invariant covariates, which allows a sensitive analysis of the determinants of a specific event, in this case, divorce. Time-varying variables included number of siblings and offspring; and time-invariant covariates included postmarital residence (categorical variable), duration of trial marriage (continuous variable), marriage cohort (categorical variable), age at marriage (categorical variable), and presence of dowry and bridewealth (categorical variable). Each person-year represents the possibility of an event, starting from the time when the marriage was initiated, and ending either with the year they got divorced or censored at the end of the observation period (in the year 2015 or in 2016, depending on the village of residence).

We used event history analysis to explore whether 1) the survival status of offspring, 2) the presence of bridewealth and dowry, and 3) the length of trial marriage were associated with the risk of divorce. Time-varying variables include the birth and the death of offspring, and time-invariant variables include the existence of siblings at the time of marriage and whether the postmarital residence was in the same village as the natal household. The age and time period at marriage for each individual, as well as the number of siblings and residential status before and after marriage, were entered as control variables (see Supplementary Table 2 for detailed descriptive statistics). Among 369 male and 420 female interviewees, there were *N* = 6206 person-years for females (and *N* = 6121 person-years for males (Supplementary Information); the event of divorce was *N* = 114 for females and *N* = 94 for males interviewed. Among all those who divorced, 90% of divorced females and 68% of divorced males in our sample had divorced during the first 10 years of marriage.

We used model selection to determine which variables were associated with marriage duration. The six candidate models include the control variables and combinations of the four independent variables described above: control model, control and offspring model, control and sibling model, control and trial marriage model, control and brideprice model as well as the full model. The best model was the full model based on the AICc (Supplementary Table 3). The Polr (Proportional Odds Logistic Regression) function in package MASS was used when the dependent variables were ordinal, for example, to analyze the labor rating data. All the statistical analyses were performed using R (v.3.4.4).

## RESULTS

### Predictors of divorce

The mean age at first divorce for men was 24.28 years (min age = 15.00, max age = 60.00, mean age = 24.28 ± 7.80 SD), while for women it was 20.16 years (min age = 14.00, max age = 36.00, mean age = 20.16 ± 3.37 SD). In addition, variance in age at divorce was greater among men than women, with men more likely to keep divorcing until a much older age compared to women (Supplementary Figure 3). Women were statistically more likely to stay single after their first divorce whereas men were more likely to remarry quickly (Supplementary Figure 3, chi squared = 14.629, df = 1, *P* < 0.001).

Table 1 shows the best fitting model after model selection. Regardless of offspring survival status, the presence of children was associated with a lower likelihood of divorce. Child birth (odds ratio [OR] = 0.57, 95% CI = 0.47, 0.69, *P* < 0.001) and death (OR = 0.57, 95% CI = 0.41, 0.81, *P* = 0.001) were both negatively associated with risk of divorce. Brothers were associated with their sister’s marriage duration, but older and younger brothers had effects in opposite directions. Younger brothers increased the odds of divorce (OR = 1.18, 95% CI = 0.98, 1.42, *P* = 0.08), whereas older brothers reduced the chance of their sister’s separation (OR = 0.80, 95% CI = 0.62, 1.03, *P* = 0.08).

**Table 1:** Event history analysis (EHA) on risk of divorce for women. *N* = 420 women with 6206 person-years, the event of divorce is 114. The dependent variable is whether divorced (1 = divorced, 0 = not divorced). Variables in the analysis include time-variant and time-invariant variables. Control variable includes cohort of year of marriage and age of marriage.

Variables	OR	Estimate (SE)	*P* value
Age of marriage (ref:<20)			
20–25	1.41	0.34 (0.29)	0.230
>25	0.70	−0.37 (0.33)	0.274
Time of marriage (ref:<1990)			
1990–2000	1.69	0.53 (0.33)	0.110
**2001–2015**	**2.66**	**0.98 (0.31)**	0.001**
**Trial marriage duration (years)**	**0.82**	−**0.2 (0.09)**	0.032*
**Child born (ref. no birth)**	**0.57**	−**0.56 (0.10)**	<0.001***
**Child death (ref: no death)**	**0.57**	−**0.56 (0.18)**	0.001**
**No. older brother(continuous)**	**0.80**	−**0.23 (0.13)**	0.081*
No. older sister(continuous)	0.97	−0.03 (0.12)	0.827
**No. younger brother**(continuous)	**1.18**	**0.16 (0.10)**	0.083*
No. younger sister(continuous)	0.95	−0.06 (0.12)	0.675
**Birthplace (ref: natal)**	**1.91**	**0.66 (0.21)**	0.002**
**Dowry (ref: no dowry)**	**0.66**	−**0.42 (0.23)**	0.064*
**Bridewealth (ref: no bridewealth)**	**0.62**	−**0.47 (0.28)**	0.087*

OR, odds ratio; SE, standard error. Statistically significant effects are indicated in bold.

*P < 0.05, **P < 0.01, ***P < 0.001.

Trial marriage duration, modeled as a continuous variable, was an important predictor of the duration of the marriage (Min year = 2.00, Max year = 11.00, Mean year = 2.725 ± 1.455 SD); the longer the trial time before marriage, the more likely the marriage was to survive (OR =0.82, 95% CI = 0.69, 0.98, *P* =0.032). Cohort also had an important influence as the proportion of marriages preceded by a trial mating system has reduced over recent years (Supplementary Figure 4). As we can see from Table 1, the risk of divorce increases over time from 1990 onwards, especially for the marriages after 2000, when the odds of divorce were 2.66 times higher than for marriages started before 1990.

**Figure 3:**
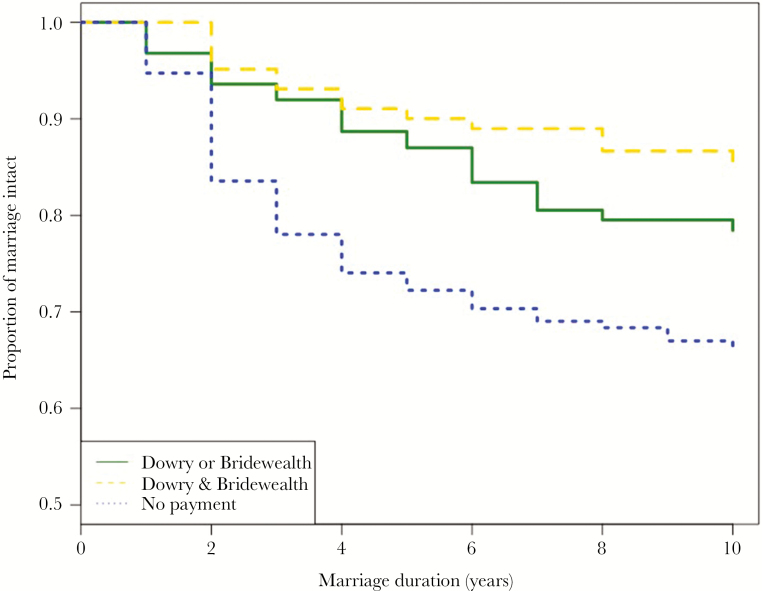
The probability of divorce during the first 10 years of marriage. The Kaplan–Meier curve shows the survival probability of the marriage, yellow dash line indicates women have both bridewealth and dowry in her marriage, green line indicates that women have either dowry or bridewealth, and blue dots show that there is no marriage payment.

As detailed above, following the collapse of the commune system, there was a return to a system of family wealth, bridewealth or dowry (at the time of marriage) were transferred to offspring by parents. Figure 1 shows the distribution of bridewealth and dowry, based on the number of yaks given. Dowry (Min = 0.00, Max = 80.00, Mean = 12.06 ± 15.71 SD) was significantly larger than bridewealth (Min = 0.00, Max = 105.00, Mean = 6.87 ± 14.74 SD) (Welch *t*-test, *P* = 0.006). For females, dowry payments from the bride’s family significantly reduced the risk of divorce (OR = 0.66, 95% CI = 0.42, 1.02, *P* = 0.064), as did bridewealth payments from the groom’s family (OR = 0.62, 95% CI = 0.36, 1.07, *P* = 0.08). When neither side of the family contributed to the “common pool” of marriage payments, the risk of divorce was highest (Figure 3). Women were also more likely to get divorced if their natal house was in the same village as their postmarital residence (Table 1; OR = 1.91, 95% CI = 1.26, 2.88, *P* = 0.002). This may be because women are more likely to go back to their natal household to seek help from parents or siblings if the natal house is close by.

### Reputation for hard work

**Figure 4:**
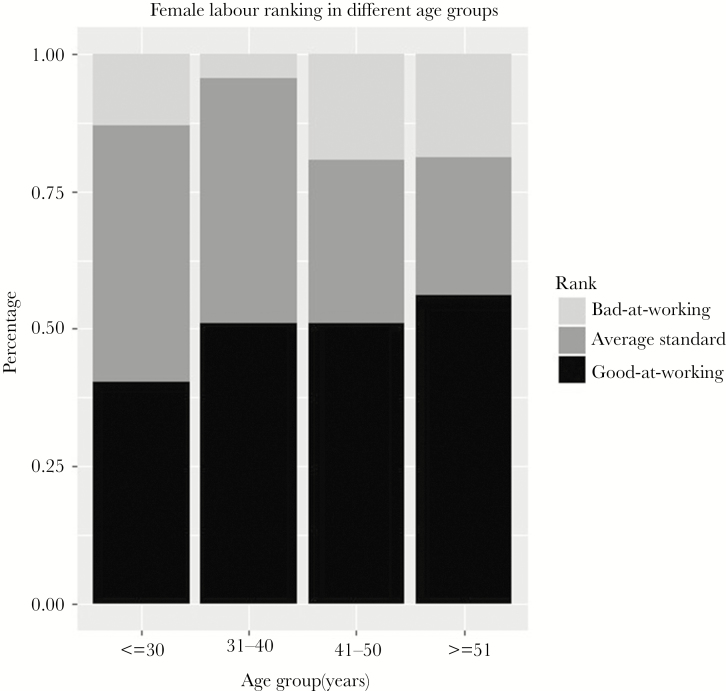
Women’s working ability rates, split by age group as ranked by other villagers. Black bar means those ranked “good-at-working” women, dark gray bar means women who are ranked at a medium standard of working ability, and light gray bar means those women ranked as “bad-at-working.”

Table 2 shows factors associated with ratings for hard work among women, controlling for women’s wealth, age, and the total number of offspring (mean number of animals = 86.71 ± 50.63 SD, the mean number of children = 3.5 ± 2.05 SD). Age had little effect on the ratings in the sense that women in late middle age were rated as more hard working than were those under 30 (Figure 4). Wealthy women (i.e., more yaks owned by the family) were less likely to be rated as bad workers. The variable “marital status of women” codes whether a woman has ever been divorced or not. As we can see from the proportional odds ratio in Table 2, when a woman’s marital status changed from married to divorced the odds of receiving a low rating increased to 2.48 (so from the rating of “good at working” to “average,” or from “average” to “bad at working”). We cannot make strong claims about the directions of causality, that is, whether this was because they are less hard working and so are more likely to be divorced, or whether it is because divorced people are more likely to be considered to be bad workers. We would suggest, however, that the negative association between a women’s marital status and her labor rating works in both directions.

**Table 2:** Covariates associated with labor rating of women (high values indicate those rated as harder workers)

Parameters	OR	SE	*t* value	*P* value
No. yaks	0.99	0.002	−2.06	0.039
Age	0.99	0.159	−0.511	0.609
No. children	0.90	0.101	−0.994	0.320
Marital status (ref: never. divorced)				
**Divorced**	**2.48**	**0.375**	**2.421**	0.015**

OR, odds ratio; SE, standard error; statistically significant effects are indicated in bold.

*P < 0.05, **P < 0.01, ***P < 0.001.

## DISCUSSION

Our results were in line with our predictions. Children were associated with an increase in marital stability, as was the payment of bridewealth and dowry, and the presence of trial marriage. A lack of material investment in the marriage was associated with a greater risk of divorce, and divorced women were more likely to be considered bad workers, suggesting that reduced investment of labor also increased divorce risk. Women were less likely to remarry following divorce than men. These results probably reflect the fact that the female labor contribution to the household is substantial and therefore key to the family’s economic development (Du and Mace 2018).

We also found that divorce risk has increased over time. This is true in many modern societies (Glick and Lin 1986; Amato 2000). In Chinese society, the government policy changes in the 1990s may have influenced this trend. Reproductive policy has limited fertility to three children per woman in our study area since the early 1990s. Some women are therefore sterilized during their twenties, as they already have three children by this age. This could possibly have increased divorce risk, as men can still reproduce in future marriages.

Another explanation for increased rates of divorce may relate to women’s capacity to raise children successfully outside of marriage. After 1990, land privatization was also implemented, and women came to own their own portions of land. Although a bride is not able to bring land into the marriage as dowry, she will usually receive payment from her family per year as land rental fees. Thus, land rights and the resulting income for women may also have increased the possibilities for women to divorce. Marlowe (2000) has argued that, in pastoralist societies, males provide more resources to the family but put less effort into direct child care compared to males in other subsistence societies (Marlowe 2000). In this study, we did not observe males investing heavily in food production, which appears to be seen as mostly women’s work. A woman’s invaluable economic contribution to the family also means that sisters are popular among their unmarried younger brothers, and may influence a woman’s chances of being welcomed back into her natal home if she is not happy in her marriage. There are many divorced women in this community who have not officially remarried, and are raising children as single parents, often with the support of their natal families. Some women may also be in unofficial polygynous relationships with married men and may receive help from them. As the division of labor in this system requires only low input from males, it seems that women can survive and raise children relatively successfully inside or outside the institution of marriage.

Another reason for increasing divorce rates in this Tibetan society may be the reduced rate of trial marriage. Trial marriage is important: women normally reside virilocally during the trial marriage, giving the male partner and parents-in-law time to evaluate the fertility and working ability of the potential bride. The economic and fitness-related benefits of trial marriage for a female partner are less clear. We cannot say whether it is the man or his family who takes the initiative to divorce a woman, or whether greater economic independence for women gives them more confidence to leave if they are not happy, but removing trial marriage reduces the chances of informed partner choice before marriage.

Marriage is by far the most important act of partner choice in most people’s lives. Here, we have documented a case which illustrates how that partnership is more likely to survive when 1) partners first have the opportunity to assess the quality of their potential spouse before marriage and 2) have an initial investment of wealth in the form of marriage transfers from both the families that are party to the union. However, investment in the union after marriage appears unequal from the man and woman. This unequal division of labor may partly reflect recent changes in the history of the area; men are no longer much needed for their traditional roles in resource defense, raiding or for active herding, given the fenced pastures; and cultural institutions can be slow to change (Du and Mace 2018). Alternatively, it may reflect the biased operational sex ratio, generated at least in part by the monastic system. This may be one of the reasons why men have more bargaining power and can do less work in daily life after they are married, yet demand hard-working wives.

In this research, we used detailed demographic data in a pastoralist society, where marital status is diverse, to investigate factors associated with divorce from a behavioral ecology perspective but there are questions that warrant future work. For instance, although we think sex ratio is an important factor, we do not have longitudinal data to support this argument. In addition, we only looked at the first marriage, and it would be interesting to have further within-subject analysis as this would enable the respondents to act as their own control with respect to the influence of factors like trial marriage and the level of bridewealth and dowry.

## FUNDING

This work was supported by the China postdoctoral grant and the Fundamental Research Funds for the Central Universities (Grant No. 505000-561219034).

## Supplementary Material

arz115_suppl_Supplementary_Material
