# Supplementary material for: Marriage stability in a pastoralist society
Source: Behav Ecol. 2019 Jul 8;30(6):1567–74. doi: 10.1093/beheco/arz115 (PMC6838654; doi:10.1093/beheco/arz115)
Supplement: arz115_suppl_Supplementary_Material [file arz115_suppl_supplementary_material.docx]

Supplementary Information for

Marital stability in a pastoralist society

Ethnographical setting

Because of the ecology in rural Amdo Tibetan areas, “dating” is almost impossible for young people; especially women are always busy doing herding and housework in their natal homes and the population density is low, so that the chance for women and men meet each other is small. Most often partner selection was arranged by parents or relatives in the past, but with the development of the technology, young people have more freedom to communicate with each other through phones, but parents’ opinion is also important. After several occasions of seeing each other, the man and woman will start to live together and enter into a “trial marriage”. Trial marriage usually starts with living together in the man’s parents’ house. Before formal marriage, there is no proper house or tent for the new couple to stay in. Parents’ opinion is very important in making a decision on whether to bring a female into the family, because of her ability to work means a lot for the prosperity of the family. Before cohabitation begins, after briefly talking to each other on the phone, one day deep in the evening, the girl will disappear from her natal house (planned beforehand secretly), and the man will bring her to his parents’ house. The next morning, the man’s family will find this new family member. Most often, the men’s parents will bring some small gifts (tea, soft drinks, white scarf etc.) to the girl’s family the next morning to show respect, but some parents will wait for several days until they are also satisfied with their potential daughter-in-law. When a man and a woman start living together, she starts to work for her husband-to-be's family, until one day the trial process ends and the two families will move on to the next stage, where the wedding ceremony will be organized and details of marriage discussed. Most importantly the exchange of bridewealth and dowry will proceed. There are also a few cases where, after several years of trial marriage, a woman who doesn’t get pregnant is abandoned by the man’s family and she will go back to her natal house and wait for the next mating opportunity. But she will have a bad reputation in the small-scale society, at risk of being considered to be barren or lazy. Parents can also arrange a marriage for their children, and in this case, the whole process will become simpler and sometimes they will skip the trial procedure.

**SI Figure 1:** Marital status of women and men in the sample. X-axis is the marital status, Y-axis is frequency. ‘Unmarried’ means adults who never married but do have children. ‘Stay divorce’ means after divorce they have not married again. Blue bar indicates men, pink bar indicates women.


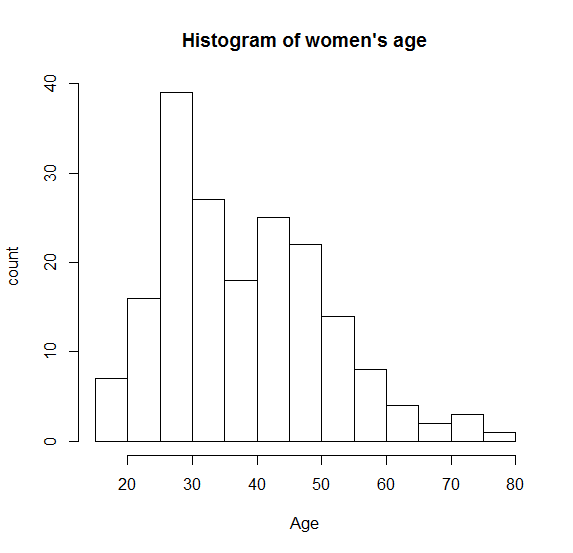


**SI Figure 2**: Age distribution of women in the sample whose labour ability were rated.


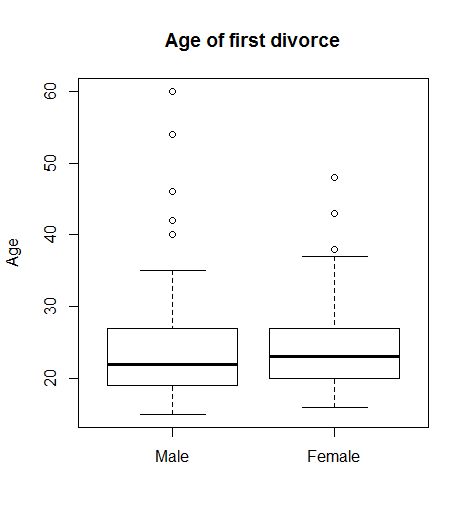


**SI Figure 3**: Boxplot of first divorce age for males and females.

**SI Figure 4**: Trial Marriage Change Through Time. N=420 female marital information was used in the plot. X-axis is the time of marriage, Y-axis is frequency shown as a proportion. Blue bar indicates no trial marriage, orange bar indicates that there is trial marriage before the formal wedding.

| Sex ratio | Whole population | Excluding monks |
| --- | --- | --- |
| Sex ratio at birth | 99.51 | 99.51 |
| Operational sex ratio (15-50) | 98.82 | 90.48 |
| Population sex ratio | 97.04 | 89.83 |

**SI Table 1**: Sex ratios of the living population with and without monks. Sex ratio at birth was calculated for the living male birth to female birth; Adult sex ratio was calculated for the adults aged 15-50; Population sex ratio was calculated from all living males to females.

| **Female Marriage** |  |  |  |  |  |  |
| --- | --- | --- | --- | --- | --- | --- |
|  | Not divorced |  |  | Divorced |  |  |
| Variables (units) | Range | Mean | SD | Range | Mean | SD |
| Year of Marriage (factor) | (1960, 2015) | 2001 | 11.214 | (1975, 2015) | 2001 | 9.606 |
| Trial time in years (numeric) | (2, 11) | 3.037 | 1.697 | (2, 6) | 2.575 | 0.914 |
| Living children (factor) | (0, 11) | 2.826 | 2.023 | (0, 5) | 1.289 | 1.158 |
| Dead children (factor) | (0, 8) | 0.639 | 1.016 | (0, 4) | 0.211 | 0.602 |
| Number of older brother (numeric) | (0, 6) | 0.496 | 0,901 | (0, 5) | 0.465 | 0.904 |
| Number of older sister (numeric) | (0, 6) | 0.431 | 0.902 | (0, 4) | 0.509 | 0.914 |
| Number of younger brother (numeric) | (0, 6) | 0.545 | 1.064 | (0, 5) | 0.702 | 1.113 |
| Number of younger sister (numeric) | (0, 4) | 0.494 | 0.904 | (0, 3) | 0.544 | 0.894 |
| Birthplace (factor) | (0, 1) | 0.415 | 0.493 | (0, 1) | 0.628 | 0.485 |
| Size of bridewealth in yaks (numeric) | (2, 68) | 11.97 | 17.277 | (2, 62) | 5.519 | 10.169 |
| Size of dowry in yaks (numeric) | (2, 63) | 14.66 | 16.279 | (2, 61) | 9.369 | 14.899 |
| **Male Marriage** |  |  |  |  |  |  |
|  | Not divorced |  |  | Divorced |  |  |
| Variables (units) | Range | Mean | SD | Range | Mean | SD |
| Year of Marriage (factor) | (1947, 2015) | 2001 | 11.281 | (1948, 2013) | 1997 | 10.868 |
| Trial time in years? (numeric) | (2, 11) | 2.395 | 1.077 | (2, 6) | 2.259 | 0.639 |
| Living children (factor) | (0, 12) | 3.084 | 2.297 | (0, 6) | 0.977 | 1.291 |
| Dead children (factor) | (0, 8) | 0.654 | 1.07 | (0, 3) | 0.271 | 0.625 |
| Number of older brother (numeric) | (0, 5) | 0.573 | 1.013 | (0, 5) | 0.553 | 0.994 |
| Number of older sister (numeric) | (0, 7) | 0.632 | 1.116 | (0, 5) | 0.612 | 1.059 |
| Number of younger brother (numeric) | (0, 5) | 0.574 | 1.073 | (0, 4) | 0.435 | 0.823 |
| Number of younger sister (numeric) | (0, 6) | 0.447 | 0.922 | (0, 4) | 0.518 | 0.895 |
| Birthplace (factor) | (0, 1) | 1.772 | 0.419 | (0,1) | 0.682 | 0.468 |
| Size of bridewealth in yaks (numeric) | (2, 68) | 13.01 | 17.125 | (2, 55) | 4.705 | 8.312 |
| Size of dowry in yaks (numeric) | (2, 63) | 15.75 | 17.091 | (2, 60) | 8.474 | 14.526 |

**SI Table 2**: Descriptive data of female marriage. The table was divided by the event equals to 1 (divorced), and event equals to 0 (not divorced).

| **Models** | **LogLik** | **AICc** | **delta** | **weight** |
| --- | --- | --- | --- | --- |
| Full model | -483.479 | 999.0 | 0.00 | 1 |
| Control+offspring | -500.518 | 1017.1 | 18.01 | 0 |
| Control+trial | -520.478 | 1055.0 | 55.93 | 0 |
| Control+brideprice | -524.065 | 1066.2 | 67.11 | 0 |
| Control | -529.444 | 1070.9 | 71.85 | 0 |
| Control+sibling | -526.947 | 1073.9 | 74.88 | 0 |

**SI Table 3**: Model selection for the odds of divorce for female. Candidate models were ranked in ascending order based on the AICc.

| **Variables** | **OR** | **Estimate(SE)** | **P value** |
| --- | --- | --- | --- |
| Event of divorce for Female (ref: Male) | 1.111 | 0.106（0.198） | 0.594 |
| Age of marriage (ref: <20) |  |  |  |
| 20-25 | 0.938 | 0.064（0.199） | 0.749 |
| **>25** | **0.407** | **0.899（0.237）** | **<0.001***** |
| **Time of marriage (ref:<1990)** |  |  |  |
| **1990-2000** | **1.822** | **0.600（0.226）** | **0.008**** |
| **2001-2015** | **2.415** | **0.881（0.209）** | **<0.001***** |
| **Trial marriage duration(continuous)** | **0.853** | **0.159（0.076）** | **0.036*** |
| **Child born (ref: no birth)** | **0.545** | **0.606（0.073）** | **<0.001***** |
| **Child death (ref:no death)** | **0.643** | **0.442（0.121）** | **<0.001***** |
| Older brother (continuous) | 1.019 | 0.019（0.121） | 0.877 |
| Older sister (continuous) | 0.984 | 0.016（0.111） | 0.884 |
| Younger brother (continuous) | 0.849 | 0.163（0.143） | 0.255 |
| Younger sister (continuous) | 1.063 | 0.061（0.128） | 0.633 |
| Birthplace (ref:natal) | 1.359 | 0.307（0.163） | 0.059. |
| **Dowry (ref: no dowry)** | **0.428** | **0.849（0.168）** | **<0.001***** |
| **Bridewealth (ref: no bridewealth)** | **0.576** | **0.552（0.214）** | **0.009**** |
| Female:Obro(Male:Obro) | 0.837 | 0.178（0.174） | 0.306 |
| Female:Osis(Male:Osis) | 0.988 | 0.122（0.159） | 0.939 |
| Female:Ybro(Male:Ybro) | 0.391 | 0.330（0.173） | 0.056. |
| Female:Ysis(Male:Ysis) | 0.939 | 0.063（0.178） | 0.723 |

SI Table 4: Results of EHA on the risk of divorce (N= 420 women with 6206 person-years, the event of divorce is 114; N= 369 men with 6121 person-years, the event of divorce is N=94).
